# Draft Genome Sequence of *Bradyrhizobium* sp. Strain LVM 105, a Nitrogen-Fixing Symbiont of *Chamaecrista fasciculata* (Michx.) Greene

**DOI:** 10.1128/MRA.00132-19

**Published:** 2019-04-04

**Authors:** Hari B. Krishnan, Won-Seok Kim, Scott A. Givan

**Affiliations:** a Plant Genetics Research, USDA-Agricultural Research Service, Columbia, Missouri, USA; b Plant Science Division, University of Missouri, Columbia, Missouri, USA; c Informatics Research Core Facility, Molecular Microbiology and Immunology, University of Missouri, Columbia, Missouri, USA; University of Arizona

## Abstract

Here, we present the draft genome sequence of Bradyrhizobium sp. strain LVM 105, a soil bacterium that forms nitrogen-fixing nodules on the roots of partridge pea. The genome size was 8,386,213 bp, with a GC content of 63.8%. The draft assembly contained 97 scaffolds with an *N*_50_ value of 415,026 bp.

## Abstract (precis)

Here, we present the draft genome sequence of Bradyrhizobium sp. strain LVM 105, a soil bacterium that forms nitrogen-fixing nodules on the roots of partridge pea.

## ANNOUNCEMENT

Partridge pea, Chamaecrista fasciculata, is an annual legume belonging to the subfamily Caesalpinioideae. This legume enters into symbiosis with rhizobia, resulting in the formation of nitrogen-fixing nodules. The nodulation process in Chamaecrista is believed to have evolved independently from that of the subfamily Papilionoideae, representing around 60 million years of independent evolution (1, 2). The characterization of the genomes of the symbionts will afford insights into the origin and evolution of nitrogen-fixing nodules. Here, we present the sequence assembly and annotation of a rhizobium that forms nitrogen-fixing nodules in partridge pea.

*Bradyrhizobium* sp. strain LVM 105 was isolated from nodules of partridge pea growing in a wooded region in Columbia, Missouri (USA), following established protocol (3). A single colony was grown in liquid yeast extract mannitol (YEM) medium (55 mM mannitol, 0.1% yeast extract, 3 mM K_2_HPO_4_, 0.8 mM MgSO_4_, and 1.7 mM NaCl; pH 6.8) for 4 days at 30°C. Genomic DNA was isolated using DNAzol reagent (Thermo Fisher Scientific, Waltham, MA) and sheared to generate average fragment sizes of 550 bp. A library was constructed following the manufacturer’s protocol with reagents supplied in the TruSeq DNA PCR-Free sample preparation kit (Illumina, San Diego, CA). The library was diluted and sequenced according to Illumina’s standard sequencing protocol for the MiSeq platform. To assemble the draft genome sequence, we started with a single 150-mer paired-end Illumina MiSeq data set derived from a single shotgun library. Using relatively light settings (minimum base quality of 15 and minimum read length of 125), 1,305,481 forward and reverse reads (2,610,962 total reads) were trimmed and filtered to remove low-quality bases using the FASTX-Toolkit (http://hannonlab.cshl.edu/fastx_toolkit/index.html). Reads were also filtered to remove PhiX using Bowtie version 1.2.2 (4). Subsequent to the quality control regimen, 1,070,398 properly paired reads and 188,147 unpaired reads were assembled using SPAdes version 3.11.1 using the “-k auto” option flag (5, 6) and the BayesHammer error-correction algorithm. Actual kmer lengths selected during the assembly were 21, 33, 55, and 77. After removing contigs shorter than 500 nt in length, the final assembly contained 97 scaffolds with a total length of 8,386,213 nt, with an *N*_50_ value of 415,026 nt and a GC content of 63.81%. We used a variety of software to evaluate the resulting assembly, including QUAST (7), REAPR (8), FRC (9), and custom Bash scripts. The mean read coverage for the assembly was 37.82×. The protein-coding open reading frames were predicted and annotated using the NCBI Prokaryotic Genome Annotation Pipeline (10).

A total of 7,453 protein-coding sequences, 3 rRNA genes, and 51 tRNA genes were detected. A BLAST search analysis of the 16S rRNA gene sequence of LVM 105 revealed close phylogenetic relationships with different strains of Bradyrhizobium diazoefficiens. Genes involved in motility, root colonization, and siderophore production were found in the genome. Genes involved in nitrogen fixation and nodulation were also detected. A cluster of genes coding for a type III secretion system (11), which is involved in the delivery of effector proteins directly into the host plants, was present in the genome.

### Data availability.

The draft genome sequence of *Bradyrhizobium* sp. strain LVM 105 has been deposited in GenBank under the accession number QZMV00000000. Raw sequence reads were deposited in the Sequence Read Archive under BioProject number PRJNA492229. The version described in this paper is the first version, QZMV01000000.
